# Comparison of MyDispense with in-person simulation in drug information training of pharmacy curriculum – a randomized cross-over study

**DOI:** 10.1186/s12909-025-06921-5

**Published:** 2025-03-09

**Authors:** Palanisamy Amirthalingam, Shahul Hameed Pakkir Mohamed, Vinoth Prabhu Veeramani, Mathar Mohideen Nagoor Thangam, Majed Falah Alanazi, Muralikrishnan Dhanasekaran, Vasudevan Mani, Kousalya Prabahar, Mostafa A. Sayed Ali

**Affiliations:** 1 Department of Pharmacy Practice, Faculty of Pharmacy, University of Tabuk, Tabuk, Saudi Arabia; 2 Department of Health Rehabilitation Sciences, Faculty of Applied Medical Sciences, University of Tabuk, Tabuk, Saudi Arabia; 3 Saveetha College of Physiotherapy, Saveetha Institute of Medical and Technical Sciences (Deemed to the University), Chennai, Tamil Nadu India; 4 Department of Medical-Surgical Nursing, Faculty of Nursing, University of Tabuk, Kingdom of Saudi Arabia, Tabuk, Saudi Arabia; 5 Department of Drug Discovery & Development, Harrison College of Pharmacy, Auburn University, Auburn, USA; 6 Department of Pharmacology and Toxicology, College of Pharmacy, Qassim University, Buraydah, 51452 Saudi Arabia

**Keywords:** Drug information, MyDispense, Student success, Student response, MD

## Abstract

**Background:**

Drug information training is restricted to pharmacy students due to the lack of opportunities for training and the inadequate number of drug information centers. Drug information simulation requires manpower and is time-consuming to arrange. MyDispense (MD) is widely accepted by numerous pharmacy schools and plays a major role in training students for various courses in the pharmacy curriculum. However, the students’ performances and perceptions of MD in drug information training involving nonjudgmental queries are yet to be established.

**Objective:**

To compare the student’s success and perceptions of virtual versus in-person simulation in providing drug information.

**Methods:**

A cross-over study design was used to compare student performance and perception of virtual and in-person simulation. A total of one hundred and forty-nine students consented to participate in the study. They were randomly allocated, with 75 assigned to the MD group and 74 to the in-person simulation group for exercise (1) Then, the students crossed over to in-person simulations and MD for exercise (2) A 5-point Likert scale questionnaire consisting of ten items was developed and validated to assess their perception regarding the learning experience of drug information exercises. Simple logistic regression was used to compare the students’ success rate, and the mean value of students’ responses was compared using non-parametric tests.

**Results:**

In exercise 1, a significant association of student success with MD was observed with task 2 (Identify the patient’s background; *p* = 0.001) and task 3 (Identify background information of the drug information query; *p* = 0.002). The students expressed a significantly higher confidence level (*p* = 0.000) when dealing with virtual patients, as reflected in their success rate regarding the identification of the background of the patient and the nature of the drug information question during exercise 1. However, students’ responses to the item related to the confidence level had no significant difference in exercise 2 (*p* = 0.382). Further, in-person and MDs had no significant differences regarding student perception of the remaining tasks in both exercises. Also, the student performances were comparable between virtual and in-person simulation in exercise 2.

**Conclusion:**

The students’ performances and perceptions were comparable between virtual and in-person simulations at different times. Therefore, MD can be implemented to train the students regarding drug information services and handle nonjudgmental queries at community pharmacies.

**Supplementary Information:**

The online version contains supplementary material available at 10.1186/s12909-025-06921-5.

## Introduction

Drug information service (DIS) is one of the responsibilities of pharmacists, and it requires various skills, including patient communication, searching for answers to drug information queries using resources, literature review, documentation, and quality assessment [1]. According to the WHO, the provision of DIS is one of the essential interventions to promote rational drug use [2]. In community pharmacies, the pharmacist usually counsels the patients about their drugs, including their names, doses, indications, time of use, and storage. Additionally, the pharmacist handles other queries relevant to over-the-counter and prescription medicines raised by the patients or visitors at the community pharmacy [3]. Therefore, the drug-related hazards to the patients were minimized during their self-medication practices at community pharmacies [4]. Recent studies reported that some pharmacists lack drug information skills due to their poor knowledge of medications, including indication, dose, frequency, duration, route of administration, missing doses, drug interactions, precautions, side effects, and storage conditions. This inadequate knowledge of some pharmacists regarding drugs restricts them from providing satisfactory patient counseling. Hence, the pharmacist should have comprehensive knowledge to provide quality DIS [5, 6]. Previous researchers have already addressed that the lack of drug information training in pharmacy curriculums for students is insufficient for pharmacy practice [7].

Lack of ability to provide DIS was already reported as the most significant challenge for community pharmacists, which can be rectified by providing adequate training during their graduation [8, 9]. In this context, preparing pharmacy students to overcome these challenges is crucial and can be achieved by introducing a practical training module for teaching drug information courses [10]. Simulation-based teaching helps students enhance their knowledge of clinical decision-making and improve their competence in real-world drug information services [11]. Also, it equips students with various drug information skills for different scenarios in a given period [12]. However, it faces several challenges, including financial and time constraints and a lack of faculty members. Therefore, pharmacy institutions must eliminate these barriers to provide quality drug information training [13].

The recently introduced virtual pharmacy simulation, the MyDispense (MD), minimizes these challenges. It is a virtual platform that helps educators train their students in pharmacy practice setups [14]. Pharmacy students were highly satisfied with this platform while learning pharmacy practice courses regarding patient interviews, dispensing, medication labeling, and patient counseling [15]. Previous studies reported the successful incorporation of the MD into the courses of therapeutics, self-care therapeutics, and pharmacy law in the pharmacy curriculum [16–18]. Additionally, other studies reported that students achieved comparable scores using the MD compared to the in-person simulation in pharmacy practice and usual therapeutic instruction [15, 16]. Although the MD platform minimizes cost, workload of faculty members, and time compared to in-person simulation, investigations regarding teaching and/or assessing drug information skills in a pharmacy school are scarce. Therefore, the present study was pioneered to explore the students’ performances and perceptions of introducing MD in drug information courses by comparing it with in-person simulation using a randomized cross-over study design.

## Methods

### Concept

The fifth-year Pharm.D. curriculum comprises the drug information course, which trains students to systematically answer drug information queries by searching various resources. According to one of the course learning objectives, the exercises are usually given in simulation training and consist of exercises with nonjudgmental queries. The example for a nonjudgmental query has been attached as a supplementary file 1. The students should answer the queries using relevant resources within a given time. In-person simulation is an actual training method in drug information practice in our curriculum that requires a lot of manpower and is time-consuming to arrange for a standardized patient. MD is widely used in pharmacy practice courses and successfully in our curriculum, using the MyDispense online database established by Monash University Australia [14]. This study attempts to investigate the comparison of MD with in-person simulation in a drug information training.

### Study design and ethical considerations

**Fig. 1:**
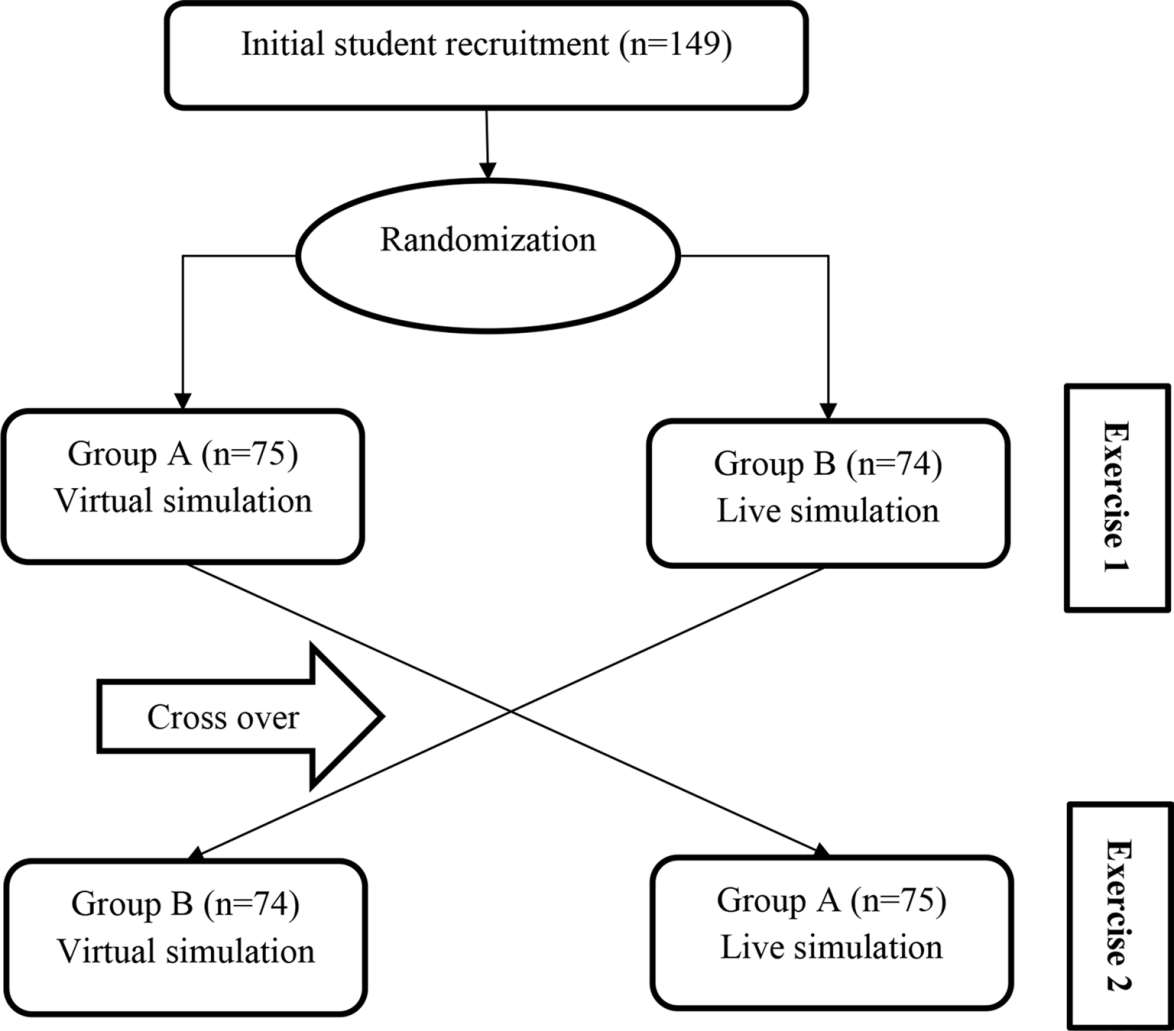
Cross-over study design for the student recruitment process in exercises 1 and 2

A randomized crossover study compared the student scores and perception between the virtual and in-person simulations (Fig. 1). Our university’s local research ethics committee approved the study, and informed consent was obtained from all the students involved.

### Students recruitment and study process

A simple random sampling method was used, and all one hundred and forty-nine students in the fifth year, Pharm.D., consented and were given gift cards to participate in the assessment and survey (Fig. 1). The students were randomly divided into groups A (*n* = 75) and B (*n* = 74). Groups A and B were assigned to virtual and in-person simulations, respectively. Both groups were given exercise 1 simultaneously and asked to respond to the questionnaire after completing the exercise. Then, the groups were crossed over for exercise 2. The student’s response to the 5-point Likert scale questionnaire was documented immediately after completing the exercise.

### Details of the exercise

The approach for answering drug information queries was similar in the virtual and in-person simulations (Table 1). The learning objective was identical to exercises 1 and 2. The students were already well-experienced in using MDs in other pharmacy practice courses for patient interviews, drug dispensing, and patient counseling. They had also been exposed to standardized patients regarding patient interviews and counseling in pharmacy practice courses. However, the course instructors provided all the students with in-person and MD demonstrations.

**Table 1:** Details of steps involved in answering drug information queries in a simulated hospital pharmacy

Steps	Task	Approach	Simulation	
			Virtual	In-person
1. Receipt of drug information query	Patients visiting the pharmacy with drug information queries and students can ask relevant questions to 1. Understand drug information query 2. Identify the background of the patient 3. Identify background information of the drug information query 4. Determine and categorize the actual question	Interactive approach	Patient fact-finding • Patient image will appear on the screen, and students should ask relevant questions	Patient interview • The student should ask relevant questions to the standardized patient
2. Searching answers using drug information resources and preparing answers for drug information query	5. Select appropriate resources for searching for answers (Similar in both the simulations) 6. To prepare the correct answer	Non-interactive approach	a. Tertiary resources • Electronic copies of textbooks, databases, and relevant websites b. Answers need to be written in the Word file	a. Tertiary resources • Electronic copies of textbooks, databases, and relevant websites b. Answers need to be written in the Word file
3. Answering drug information query	7. Answering for DI query 8. Follow-up questions • To verify the patient’s understanding and satisfaction	Interactive approach	Answers for drug information and counseling notes to be written in the patient counseling section	Live interaction with the standardized patients
4. Documentation	9. Documentation form must be completed	Non-interactive approach	Documentation form to be filled in a Microsoft Word file and to be uploaded in attachments	Documentation form to be filled manually and attached with the exam
5. Time management	10. Has to complete within the given time	Non-interactive approach	-	-

### Study site

The in-person simulation was conducted in a simulated pharmacy, and the MD was a web-based exercise using the MyDispense database performed in a computer lab. In the in-person simulation, standardized patients acted as patients visiting the community pharmacy with drug-related queries.

### Case scenarios

The course instructors designed case scenarios, such as ‘The patient is seeking drug information from the community pharmacist.’ The students were asked to answer the patient’s questions regarding the dosing schedule, drug administration, side effects, and the consequences of missing doses. Two colleagues in our University’s Department of Pharmacy Practice, Faculty of Pharmacy, peer-reviewed the case scenarios. They assessed them using reliability statistics with the desirable Cronbach’s α (> 0.8) for both item and scale statistics [19, 20]. The outcome of the case scenarios was carefully assessed in a pilot study, which ensured that exercises 1 and 2 were equally poised in terms of difficulty.

### Patient

In an in-person simulation, the student can ask relevant questions to understand the purpose of the query to the standardized patient. In MD, a 3D interactive patient animation usually appears on the screen with a pre-designed case scenario, and the fact-finding option allows the student to raise relevant questions.

### Steps involved in answering drug information query

Table 1 illustrates the five steps involved in the exercise: (1) receipt of drug information query, (2) searching for answers using drug information resources and preparing answers for drug information query, (3) answering drug information query, (4) documentation, and (5) Time management [21, 22]. The first step was receiving a drug information query and an interactive approach with a standardized patient (in-person simulation) or 3D interactive patient animation (MD), which included four tasks. In the second step, the students had two tasks: they were asked to search for answers using tertiary resources, including electronic resources, textbooks, databases, and relevant websites, according to the need for the case scenario. The third step was another interactive approach with the patient, which included two tasks: the student answering the drug information query and follow-up questions from the patient. Documentation was the fourth step in the exercise to enter the patient demographics, drug information query, mode of receipt of drug information query, category of question, the time needed to provide information, way of reply, resources used, and answer for the query. At last, a maximum of 30 min was given for the students to complete each exercise, and all students were asked to conduct all the activities within this time frame.

### Assessment methods

Similar rubrics were utilized to evaluate the virtual and in-person simulations (Table 1). The grade distribution was assigned for receipt of drug information, developing a search strategy, answering drug information queries, documentation, and time management. The exercise had ten tasks, and one mark was awarded for completing each task. According to the university policy, the passing score was at least 60% (6 marks out of 10), and the students who obtained ≥ 60% were considered successful in the exercise. Also, we assessed the student’s completion of each task. The student success rate and ability to complete each task were compared between MD and in-person simulations.

### Designing and validation of questionnaire to assess feedback from the students

A 5-point Likert scale questionnaire with 10 items assessed the student perception of MD and in-person simulation. The questionnaire has ten items related to the student’s assigned tasks.

Questions from 1 to 4 associated with the receipt of drug information query.

1. I was confident during the interaction with the standardized patient,
2. I felt that the standardized patient looked like a regular patient,
3. The interaction with the standardized patient was useful in obtaining the information related to the drug information query,
4. I have no hindrances in collecting the information from standardized patients)

Questions 5 and 6 regarding their perception of resources for answering drug information queries and experience in answering drug information queries, respectively.

5. The resources were readily available for searching for the answers to drug information queries.
6. I feel that I had a good experience in preparing the answers to drug information queries with this simulation session.

Questions 7 and 8 were related to the student’s perception of communicating the answers to the standardized patient.

7. It was interesting to communicate the answers with the standardized patient.
8. Asking follow-up questions to the patients and answering them improved my skills further.

Questions 9 and 10 were about documentation and time management.

9. The documentation was comfortable in the session.
10. I had enough time to complete the exercise.

To assess student perception regarding the interaction with the standardized patient. The questionnaire was reviewed initially by two colleagues in the Department of Pharmacy Practice, and then the questionnaire was distributed to five academic experts in pharmacy practice in other institutions for content validity [19]. Item-level content validity indexes (I-CVIs) were used to determine the relevance of items and the averaging of scale-level content validity index (S-CVI/Ave) for the overall questionnaire. The scores of I-CVIs ≥ 0.78 and S-CVI/Ave ≥ 0.90 were considered excellent content validity [23]. Cronbach’s α coefficient of > 0.7 was considered acceptable internal consistency of the questionnaire [21]. The questionnaire was distributed to the students after the completion of the exercise, and the responses were documented.

### Statistical analysis

The Chi-square test was used to compare the two groups’ categorical variables of demographic characteristics and grade distribution. The students who obtained a score ≥ 60% were considered a success of the student in exercise and/or task. Simple logistic regression was used to assess the association between student success in different simulations (in-person and virtual). The unadjusted model includes only one potential determinant (student success) and dependent variable (in-person and MD) to calculate the odd ratios (OR) and confidence intervals (CI). The mean ± standard deviation (SD) scores of student responses to the 5-point Likert scale were analyzed using a Mann-Whitney U test. Also, the order effect was calculated for the same group of students between two different simulations using simple logistic regression for students’ success rates and the Wilcoxon signed-rank test for the student’s responses. In all statistical methods, *p* < 0.05 was considered statistically significant. SPSS version 25.0 was used in the statistical analysis.

## Results

### Characteristics and level of the students

Table 2 describes the characteristics and academic achievement of the students. Female students were predominant in Groups A (53.3%) and B (54%); hence, there was a statistically significant association regarding gender distribution (*p* = 0.007). In the drug information quiz, most students were found to have grade B in groups A (34.7%) and B (42%). Regardless of the groups, most students obtained Grade A in their previous in-person simulation (Group A: 41.3%; Group B: 46%) and previous MyDispense experience (Group A: 72%; Group B: 66.2%). The grade distribution between the two groups in the drug information quiz (*p* = 0.638), previous objective structured clinical examination (*p* = 0.564), and MD experience (*p* = 0.080) in the pharmacy practice course was also not significantly different.

**Table 2:** Characteristics and level of the students included in a virtual and live simulation

Demographics	Variable	Group A *n* = 75 (%)	Group B *n* = 74 (%)	*p*-value* (χ2)
Gender	Male	35 (46.7)	34 (46)	0.929 (0.007)
	Female	40 (53.3)	40 (54)	
Grade distribution in drug information quiz	A	25 (33.3)	17 (23)	0.638 (2.286)
	B	26 (34.7)	31 (42)	
	C	13 (17.3)	15 (20.3)	
	D	6 (8)	7 (9.5)	
	F	5 (6.7)	4 (5.4)	
Grade distribution in previous OSCE experience in pharmacy practice course	A	31 (41.3)	34 (46)	0.564 (2.906)
	B	26 (34.7)	24 (32.4)	
	C	15 (20)	11 (15)	
	D	1 (1.3)	4 (5.4)	
	F	2 (2.7)	1 (1.4)	
Grade distribution in previous MyDispense experience in pharmacy practice course	A	54 (72)	49 (66.2)	0.080 (8.333)
	B	14 (18.7)	10 (13.5)	
	C	2 (2.7)	11 (15)	
	D	2 (2.7)	3 (4.1)	
	F	3 (4)	1 (1.4)	

*Chi-square test; *p* < 0.05 was considered as statistically significant

### Association of student success between virtual and in-person simulation

Generally, no significant difference was observed between the two groups regarding student success rate in exercises 1 and 2 (Table 3). In exercise 1, a significant association of student success with MD was observed with task 2 (Identify the background of the patient; *p* = 0.001) and task 3 (Identify background information of the drug information query; *p* = 0.002). Grade achievement in the remaining tasks (i.e., task 1: Understand drug information query; task 4: Determine and categorize the actual question; task 5: Select appropriate resources for searching for answers; task 6: To prepare the correct answer; task 7: Answering the DI query; task 8: Follow-up questions; task 9: Documentation form must be completed; task 10: Has to complete within the given time) had no association with the type of simulations (MD and in-person). In exercise 2, there was no significant association between the simulation type and student success rate (*p* > 0.05) in all the tasks.

**Table 3:** Association of student success rate between a virtual and in-person simulation in exercises 1 and 2

Exercise 1							Exercise 2					
	The number of students who succeeded*		OR	Confidence interval		p*	The number of students who succeeded*		OR	Confidence interval		p**
	Virtual simulation (75) *n* (%)	In-person simulation (74) *n* (%)		Lower limit	Upper limit		Virtual simulation (74) *n* (%)	In-person simulation (75) *n* (%)		Lower limit	Upper limit	
**Overall**	73 (97.33)	72 (97.29)	1.013	0.139	7.393	0.989	71 (95.94)	72 (96)	1.014	0.198	5.194	0.986
Task 1	75 (100)	74 (100)	1.013	0.019	51.74	0.994	71 (95.94)	72 (96)	1.014	0.198	5.194	0.986
Task 2	72 (96)	56 (75.67)	7.714	2.163	27.502	**0.001**	71 (95.94)	70 (93.33)	0.595	0.136	2.570	0.483
Task 3	72 (96)	57 (77.02)	7.157	1.998	25.632	**0.002**	70 (94.59)	72 (96)	1.371	0.296	6.350	0.686
Task 4	72 (96)	72 (97.29)	0.666	0.108	4.109	0.662	72 (97.29)	71 (94.66)	0.493	0.087	2.777	0.422
Task 5	69 (92)	67 (90.54)	1.205	0.383	3.760	0.752	70 (94.59)	72 (96)	1.374	0.296	6.350	0.686
Task 6	71 (94.66)	72 (97.29)	0.493	0.087	2.778	0.422	71 (95.94)	72 (96)	1.014	0.198	5.194	0.986
Task 7	66 (88)	69 (93.24)	0.531	0.169	1.668	0.278	72 (97.29)	70 (93.33)	0.388	0.073	2.071	0.268
Task 8	69 (92)	72 (97.29)	0.319	0.062	1.637	0.171	70 (94.59)	71 (94.66)	1.014	0.244	4.216	0.984
Task 9	70 (93.33)	72 (97.29)	0.388	0.073	2.071	0.139	73 (98.64)	72 (96)	0.328	0.033	3.235	0.340
Task 10	72 (96)	68 (91.89)	2.117	0.509	8.805	0.302	70 (94.59)	72 (96)	1.371	0.296	6.350	0.686

*The number of students who succeeded (obtained ≥ 60% score); **p-value of simple logistic regression; OR – Odd ratio

Groups A and B examined the order effect between virtual (exercise 1) and in-person simulation (exercise 2) using simple logistic regression (Table 4). Group A was unaffected since there was no significant association with the simulation method. Overall, student success has no significant association with the simulation method; however, task 2 (Identify the patient’s background; *p* = 0.001) and task 3 (Identify background information of the drug information query; *p* = 0.004) significantly affected the student success rate.

**Table 4:** Association of student success rate between a virtual and in-person simulation in each group

Group A							Group B					
	The number of students who succeeded*		OR	Confidence interval		p**	The number of students who succeeded*		OR	Confidence interval		p**
	Exercise 1 Virtual simulation (75) *n* (%)	Exercise 2 In-person simulation (75) *n* (%)		Lower limit	Upper limit		Exercise 1 In-person simulation (74) *n* (%)	Exercise 2 Virtual simulation (74) *n* (%)		Lower limit	Upper limit	
**Overall**	73 (97.33)	72 (96)	1.520	0.246	9.373	0.651	72 (97.29)	71 (95.94)	1.521	0.246	9.378	0.651
Task 1	75 (100)	72 (96)	7.289	0.370	143.620	0.191	74 (100)	71 (95.94)	7.293	0.370	143.734	0.191
Task 2	72 (96)	70 (93.33)	1.714	0.394	7.446	0.472	56 (75.67)	71 (95.94)	0.131	0.036	0.468	**0.001**
Task 3	72 (96)	72 (96)	1.000	0.195	5.120	1.000	57 (77.02)	70 (94.59)	0.191	0.061	0.601	**0.004**
Task 4	72 (96)	71 (94.66)	1.352	0.292	6.259	0.699	72 (97.29)	72 (97.29)	1.000	0.137	7.293	1.000
Task 5	69 (92)	72 (96)	0.479	0.115	1.991	0.311	67 (90.54)	70 (94.59)	0.546	0.153	1.954	0.353
Task 6	71 (94.66)	72 (96)	0.739	0.159	3.423	0.699	72 (97.29)	71 (95.94)	1.521	0.246	9.378	0.651
Task 7	66 (88)	70 (93.33)	0.523	0.166	1.644	0.267	69 (93.24)	72 (97.29)	0.383	0.072	2.042	0.261
Task 8	69 (92)	71 (94.66)	0.647	0.175	2.396	0.515	72 (97.29)	70 (94.59)	0.486	0.086	2.739	0.413
Task 9	70 (93.33)	72 (96)	0.583	0.134	2.533	0.472	72 (97.29)	73 (98.64)	2.057	0.365	11.592	0.413
Task 10	72 (96)	72 (96)	1.000	0.195	5.120	1.000	68 (91.89)	70 (94.59)	0.647	0.175	2.396	0.515

*The number of students who succeeded (obtained ≥ 60% score); **p-value of simple logistic regression; OR – Odd ratio

### Comparison of student perception between virtual and in-person simulation

The student perception (mean) was higher in the MD regarding all the items (Table 5). However, during the interaction with the simulated patient, the confidence level was significantly higher in the MD than in the in-person simulation regarding exercise 1 (*p* = 0.000). Also, the students involved in the MD had significantly higher perceptions (*p* = 0.001) regarding the item “I had enough time to complete the exercise” than the in-person simulation in exercise (1) Meanwhile, there was no statistically significant difference between them in the survey after exercise (2) The mean value of student perception was higher in the MD regardless of the exercises in many items. Both groups perceived MD as better than in-person simulation.

**Table 5:** Students’ perception of virtual simulation and in-person simulation in exercises 1 and 2

No.	Questions	Exercise 1 Mean (SD)		*p	Exercise 2 Mean (SD)		*p
		Virtual simulation (*n* = 75)	In-person simulation (*n* = 74)		Virtual simulation (*n* = 74)	In-person simulation (*n* = 75)	
1	I was confident during the interaction with the simulated patient.	4.81 (0.44)	4.32 (0.91)	**0.000**	4.74 (0.49)	4.71 (0.58)	0.382
2	I felt that the simulated patient looked like a normal patient.	4.65 (0.60)	4.47 (0.77)	0.168	4.56 (0.73)	4.54 (0.77)	0.684
3	The interaction with the simulated patient was useful in obtaining the information related to the drug information query.	4.54 (0.71)	4.38 (0.79)	0.123	4.44 (0.79)	4.42 (0.87)	0.471
4	I have no hindrances in collecting the information from simulated patients.	4.46 (0.87)	4.42 (0.86)	0.407	4.49 (0.70)	4.35 (0.95)	0.385
5	The resources were readily available for searching for the answers to drug information queries.	4.60 (0.70)	4.36 (0.81)	0.084	4.51 (0.79)	4.44 (0.84)	0.426
6	I feel that I had a good experience in preparing the answers to drug information queries with this simulation session.	4.54 (0.71)	4.25 (0.80)	0.050	4.49 (0.70)	4.42 (0.73)	0.346
7	It was interesting to communicate the answers with the simulated patient.	4.60 (0.71)	4.36 (0.94)	0.115	4.60 (0.72)	4.40 (0.79)	0.173
8	Asking follow-up questions to the patients and answering them improved my skills further.	4.60 (0.67)	4.32 (0.82)	0.050	4.53 (0.73)	4.35 (0.95)	0.192
9	The documentation was comfortable in the session.	4.43 (0.87)	4.32 (0.85)	0.329	4.28 (1.14)	4.00 (1.11)	0.145
10	I had enough time to complete the exercise.	4.56 (0.64)	4.23 (0.93)	**0.001**	4.51 (0.79)	4.40 (0.86)	0.399

Mean (SD) value from the student response, i.e., Strongly Agree (5), Agree (4), Neutral (3), Disagree (2), and Strongly Disagree (1)

p-value of Mann-Whitney U test (Virtual Simulation vs. In-person simulation); p-value < 0.05 was considered as statistically significant

The order effect was calculated for groups A and B based on their perceptions of virtual and in-person simulation using a Wilcoxon signed-rank test (Table 6). The students belonging to group A were significantly more satisfied (*p* = 0.000) with the documentation and were comfortable in the MD simulation. Meanwhile, the students in group B had significantly higher perceptions (*p* = 0.000) regarding the item “I was confident during the interaction with the simulated patient.”

**Table 6:** Students’ perception of a virtual and in-person simulation in each group

No.	Questions	Group A Mean (SD)		*p	Group B Mean (SD)		*p
		Exercise 1 Virtual simulation (*n* = 75)	Exercise 2 In-person simulation (*n* = 75)		Exercise 1 In-person simulation (*n* = 74)	Exercise 2 Virtual simulation (*n* = 74)	
1	I was confident during the interaction with the simulated patient.	4.81 (0.44)	4.71 (0.58)	0.146	4.32 (0.91)	4.74 (0.49)	**0.000**
2	I felt that the simulated patient looked like a normal patient.	4.65 (0.60)	4.54 (0.77)	0.287	4.47 (0.77)	4.56 (0.73)	0.314
3	The interaction with the simulated patient was useful in obtaining the information related to the drug information query.	4.54 (0.71)	4.42 (0.87)	0.299	4.38 (0.79)	4.44 (0.79)	0.211
4	I have no hindrances in collecting the information from simulated patients.	4.46 (0.87)	4.35 (0.95)	0.361	4.42 (0.86)	4.49 (0.70)	0.374
5	The resources were readily available for searching for the answers to drug information queries.	4.60 (0.70)	4.44 (0.84)	0.177	4.36 (0.81)	4.51 (0.79)	0.358
6	I feel that I had a good experience in preparing the answers to drug information queries with this simulation session.	4.54 (0.71)	4.42 (0.73)	0.182	4.25 (0.80)	4.49 (0.70)	0.051
7	It was interesting to communicate the answers with the simulated patient.	4.60 (0.71)	4.40 (0.79)	0.064	4.36 (0.94)	4.60 (0.72)	0.067
8	Asking follow-up questions to the patients and answering them improved my skills further.	4.60 (0.67)	4.35 (0.95)	0.058	4.32 (0.82)	4.53 (0.73)	0.079
9	The documentation was comfortable in the session.	4.43 (0.87)	4.00 (1.11)	**0.000**	4.32 (0.85)	4.28 (1.14)	0.522
10	I had enough time to complete the exercise.	4.56 (0.64)	4.40 (0.86)	0.169	4.23 (0.93)	4.51 (0.79)	0.050

Mean (SD) value from the student response, i.e., Strongly Agree (5), Agree (4), Neutral (3), Disagree (2), and Strongly Disagree (1)

p-value of Wilcoxon signed-rank test (Virtual Simulation vs. In-person simulation); p-value < 0.05 was considered as statistically significant

## Discussion

The present study compared MyDispense and in-person simulation regarding students’ success and perception of drug information exercises by comparing two groups of students with a cross-over study design. The tasks included in this study were adopted for the first time in MyDispense to assess the student’s performance in drug information. First, in exercise 1, the student’s overall success rate was marginally higher in MD (97.33%) than in the in-person simulation (97.29%); vice versa, the student’s success rate was higher in the in-person simulation (96%) than in MD (95.94%) in exercise 2 (Table 3). Then, the different simulation methods did not affect groups A and B’s overall student success rates (Table 4). However, these differences in success rate were not statistically significant. These findings substantiate previous findings, including MD and in-person simulations, which were comparable regarding the student’s success [15, 16]. Henceforth, the MD was successfully integrated with the drug information training in the pharmacy curriculum. Meanwhile, our findings were consistent with the previous reports that the MD cannot replace the in-person simulation since there were no significant differences in overall success between MD and in-person simulation. However, MD could replace in-person simulation in pharmacy institutions with several barriers, including cost, financial constraints, or a lack of staff members [13–15].

In Table 3, exercise 1 showed a statistically significant association between the MD and students’ success rates in identifying the patient’s background (task 2) and the drug information query’s background information (task 3). This association was noted with Group B students, while Group A was unaffected (Table 4). This might be due to the order effect among the Group B students due to their decreased ability to handle standardized patients and gather information regarding the backgrounds of patients and queries. A dual point of view can address this: (1) Recruiting and training of standardized patients and (2) Students’ psychological stress to handling standardized patients. Training standardized patients is one of the key challenges in pharmacy simulation regarding acting according to the script to achieve optimal reliability. Dealing with standardized patients requires coordination, consent, practice, feedback, and mentoring, which is time-consuming [24, 25]. In this context, the course instructors encountered several challenges in optimizing standardized patients for exercises in terms of training and orientation, which required more time [26].

This reflects lower student perception in Group B students and significantly higher confidence levels while interacting with virtual patients (Table 6). The students would have found difficulties with standardized patients, and they were comfortable with the virtual patient due to an order effect. Meanwhile, group A students also had a higher perception level with standardized patients; however, there was no statistical difference in the virtual simulation (Table 6). The students were also challenged to meet the multiple standardized patients with different portrayals in different exercises, which introduced potential bias in collecting information from them [27–29]. Also, these findings substantiate the previous conclusions that MD increased student engagement and communication with virtual patients [30, 31]. Also, few studies addressed the increased confidence level of participants during their immediate interaction with the virtual patient, which was maintained for up to six months [30, 32]. This study warrants future investigations to address the effect of standardized patients on various pharmacy simulation setups.

The student success rate for the remaining tasks regarding interactive approaches (tasks 1, 4, 7, and 8) was not associated significantly, regardless of simulations and student groups (Tables 3 and 4). These results substantiate the previous findings that the student’s performances were similar while handling virtual and standardized patients [33].

Student performances had no significant association with the type of simulation in both exercises 1 and 2 regarding tasks 5 (To choose appropriate drug resource) and 6 (To prepare the correct answer). In this regard, the students’ perceptions have no statistical significance in both simulations since they could search for answers in web-based tertiary resources and electronic textbooks. Already, a previous study demonstrated that pharmacy students preferred online resources for searching for drug information [34, 35]. As noted in the present study, this might explain the improved performance and high student perception with this task. In the current study, most students found a correct answer by choosing appropriate user-friendly resources for answering drug information queries consistent with the previous finding [36].

Documentation was electronic in MD and paper-based in the in-person simulation; it had no significant association with student success (Tables 3 and 4). Meanwhile, the mean value of the student’s perception regarding ‘the documentation being more comfortable in the session’ was higher in the MD than in the in-person simulation, regardless of the exercises and simulation (Tables 5 and 6). It has already been established that MD improves pharmacy student engagement and enhances documentation performance [28].

Student perception was significantly higher in the MD regarding the time assigned to complete the exercise, and 96% of students achieved the exercise within the allocated time for exercise 1 (Table 5). In this regard, both groups had an almost equal perception of MD and in-person simulation (Table 6). This emphasizes that the students preferred MD, known for its user-friendly application that helped them complete the exercises on time [35, 36]. MD accurately assesses the time of completion, whereas in-person simulation does not. Previous researchers stated that time management was required to meet the high workload in drug information practice [37, 38]. Therefore, MD has an added advantage in training students regarding time management.

In Saudi Arabia, there is an increasing number of pharmacy schools and pharmacy graduates, which can influence the opportunity for real drug information practice experience and patient interaction, considering the limited training sites and facilities for the students [39, 40]. Virtual pharmacy simulations have the potential to address these scenarios [41], allowing students to access comparable training opportunities that may not be readily available in clinical training settings [11]. Therefore, incorporating MD into drug information training makes it worthwhile, providing additional training opportunities for the students. Furthermore, faculty members can easily upload and edit new case scenarios. Case scenarios can be allocated to a specific group of students, and the students can be evaluated quickly. Departments teaching similar courses within the curriculum have the opportunity to share common uploaded cases and customize the level of complexity and learning outcomes. This offers the advantage of reducing the workload of faculty members.

### Limitations

First, the results of the present study were obtained from only one pharmacy school with a limited number of students, which limits the generalization of the results. However, the multicentric study involving many pharmacy schools can improve the generalizability in the near future. Second, the level of students’ knowledge in handling computers varies, which might affect the results. This can be ruled out by providing adequate training in handling computers regarding MyDispense exercises and ensuring the student has no difficulties in future studies. Third, this study only focused on addressing nonjudgmental queries the patient raised. Restriction on implementing judgmental questions usually raised by healthcare professionals limits the opportunity to utilize the MD for drug information training.

## Conclusion

The students’ performances and perceptions were comparable between virtual and in-person simulations at different times. Therefore, MD can be implemented to train the students regarding drug information services and handle nonjudgmental queries at community pharmacies. It can also be used as a replacement for in-person simulation in pharmacy institutions with barriers and a lack of staff members. More studies are warranted to investigate the effectiveness of MD in answering judgmental queries.

## Electronic supplementary material

Below is the link to the electronic supplementary material.

Supplementary Material 1

## Data Availability

According to the University policy and local research ethics committee guidelines, the data should not be publicly available; however, they may be shared upon request by emailing a_hamdan@ut.edu.sa. The above-mentioned email belongs to the authorized person responsible for holding all the student-related research data in our institution.
