# Supplementary material for: Comparison of MyDispense with in-person simulation in drug information training of pharmacy curriculum – a randomized cross-over study
Source: BMC Med Educ. 2025 Mar 9;25:356. doi: 10.1186/s12909-025-06921-5 (PMC11892152; doi:10.1186/s12909-025-06921-5)
Supplement: Supplementary file 1 — Supplementary Material 1 [file 12909_2025_6921_MOESM1_ESM.docx]

**Supplementary File 1**

**Example of a non-judgemental query**

**Scenario**

Mr. O.M. has been receiving ranitidine 150 two times daily for his indigestion in your community pharmacy, and patient counseling was done by you already. However, he still has some questions regarding his medications.

**From patient to pharmacist**

- Query 1: Can I take two pills once instead of twice daily?
- Query 2: What should I do if any dose is missing?
- Query 3: Are there any side effects besides what you explained to me?
- Query 4: Should I store this medication in a refrigerator?

**Follow-up questions from the pharmacist to verify the patient's understanding**

The pharmacist must ask one of the following questions to verify the patient's understanding

Examples

- Can you please tell me how many times you will take this medication per day?
- What do you do if you miss a dose of this medication?
- How long will you take this medication?
- Where will you store this medication?
